# SdH Oscillations from the Dirac Surface State in the Fermi‐Arc Antiferromagnet NdBi

**DOI:** 10.1002/advs.202303978

**Published:** 2023-10-25

**Authors:** Ruoqi Wang, Junchao Zhang, Tian Li, Keming Chen, Zhengyu Li, Mingliang Wu, Langsheng Ling, Chuanying Xi, Kunquan Hong, Lin Miao, Shijun Yuan, Taishi Chen, Jinlan Wang

**Affiliations:** ^1^ Key Laboratory of Quantum Materials and Devices of Ministry of Education School of Physics Southeast University Nanjing 211189 China; ^2^ High Magnetic Field Laboratory Chinese Academy of Sciences Hefei 230031 China

**Keywords:** AFM spintronics, Fermi‐arc, NdBi, orbital magnetic moment, peculiar surface state, spin‐flop transition

## Abstract

The recent progress in CuMnAs and Mn3X (X = Sn, Ge, Pt) shows that antiferromagnets (AFMs) provide a promising platform for advanced spintronics device innovations. Most recently, a switchable Fermi‐arc is discovered by the ARPES technique in antiferromagnet NdBi, but the knowledge about electron‐transport property and the manipulability of the magnetic structure in NdBi is still vacant to date. In this study, SdH oscillations are successfully verified from the Dirac surface states (SSs) with 2‐dimensionality and nonzero Berry phase. Particularly, it is observed that the spin‐flop transition only appears when the external magnetical field is applied along [001] direction, and features obvious hysteresis for the first time in NdBi, which provides a powerful handle for adjusting the spin texture in NdBi. Crucially, the DFT shows the Dirac cone and the Fermi arc strongly depend on the high‐order magnetic structure of NdBi and further reveals the orbital magnetic moment of Nd plays a crucial role in fostering the peculiar SSs, leading to unveil the mystery of the band‐splitting effect and to manipulate the electronic transport, high‐effectively, in the thin film works in NdBi. It is believed that this study provides important guidance for the development of new antiferromagnet‐based spintronics devices based on cutting‐edge rare‐earth monopnictides.

## Graphical abstract

In Fermi‐arc antiferromagnet NdBi, it is first achieved 1) the SdH oscillations from the Dirac surface state with 2‐dimensionality and nonzero Berry, 2) the manipulation of the magnetic structure by spin‐flop transition with obvious hysteresis, 3) DFT revealing the peculiar surface states strongly depending on the high‐order magnetic structure and the orbital magnetic moment of Nd.

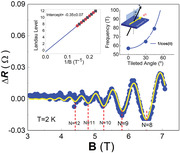

## 1 Introduction

Fermi‐arc, the segment of a closed contour in the Fermi surface, links nontrivial singularity points in momentum space and has been extensively studied in topological materials.^[^ ^1^ ^]^ Electrons riding on the Fermi‐arc lose opposite momentum wavevector under the same kinetic energy and are enforced to propagate straightforwardly without backscattering. Therefore, Fermi‐arcs interplaying with phonons, magnons, strain, etc., account for various peculiar macroscopic quantum phenomena.^[^ ^2^, ^3^, ^4^, ^5^, ^6^, ^7^, ^8^, ^9^, ^10^, ^11^, ^12^, ^13^, ^14^, ^15^, ^16^, ^17^, ^18^, ^19^, ^20^, ^21^, ^22^, ^23^, ^24^, ^25^, ^26^, ^27^, ^28^, ^29^, ^30^ ^]^ Protected by the crystal symmetry and time‐reversal symmetry breaking, Fermi‐arc exists robustly and cannot be annihilated, which has been mainly studied in nonmagnetic compounds such as TaAs family, Na3Bi, WTe2, and so on, and ferromagnetic systems such as Co3Sn2S2 and Co2MnGa, giving rise to greatly enhanced anomalous Hall conductivity, thermoelectricity, and magnetoresistivity (MR).^[^ ^1^ ^]^ Fermi‐arcs are very meaningful in developing high‐efficiency spintronic devices due to their high dependence on the chirality of magnetic charge that can be manipulated by the external magnetic field,^[^ ^11^, ^15^, ^22^ ^]^ strains in heterostructures,^[^ ^31^, ^32^ ^]^ or spin‐orbit‐torque,^[^ ^33^ ^]^ etc., thus have acquired great attention in the most recent years.

However, the switchable Fermi‐arc emerging in normal metal is rare. A recent discovery in NdBi is exciting, where the degenerate Fermi‐arcs are suddenly produced when the magnetic moments from Nd atoms are antiferromagnetically ordered at its Neel temperature of 24 K.^[^ ^34^ ^]^ Strikingly, with the temperature cooling below 20 K, the initially degenerate Fermi‐arcs begin to split into two non‐degenerate SSs, a spin‐polarized Fermi‐arc, and the helical massive Dirac cone. The nondegenerate Fermi‐arc is bent downward with a hole pocket, while the helical massive Dirac cone is curved upward with an electron pocket. The most recent work even found the peculiar SSs can be reproduced by considering high‐order magnetic structures, namely the layered collinear antiferromagnet in (001) plane (***2q***) and the 2‐in/2‐out configuration (***3q***) that is famous in the spin‐ice compound,^[^ ^6^, ^35^ ^]^ again deeply demonstrates the close relations between the peculiar electronic bands and the magnetic structure in NdBi, which could trigger a new strand of researches on antiferromagnetic spintronics. Nevertheless, from the perspective of antiferromagnetic spintronics, the magnetotransport on the new peculiar SSs, magnetic field adjusting the magnetic structure, and density functional theory (DFT) studying the SSs in NdBi are crucial, but which are still vacant to date.

Here, we report our most recent progress on this cutting‐edge issue. First, the Dirac cone SSs are successfully identified from the SdH oscillations, with the frequency of 57 T, 2‐dimensionality, nonzero Berry phase of 0.7π, and 0.23 times of free electron mass. The Fermi energy is determined to be 57 meV above the Dirac point, very close to the value evaluated from the ARPES.^[^ ^34^ ^]^ Second, our magnetization measurements show a sharp spin‐flop transition with obvious hysteresis between 2 and 4 T in NdBi, which can provide a powerful handle for controlling the peculiar SSs. Finally, our DFT calculations reveal that the peculiar SSs can be well reproduced with ***2q*** magnetic structure if Nd atoms have a larger orbital magnetic moment, which provides a key step for unveiling the mystery of the band splitting in NdBi. Our work lays the foundation for developing new spintronics devices based on the pioneer AFM compound NdBi.^[^ ^12^, ^36^, ^37^, ^38^ ^]^

## 2 Results and Discussion

### 2.1 Sample Growth and Basic Characterizations

**Figure 1:**
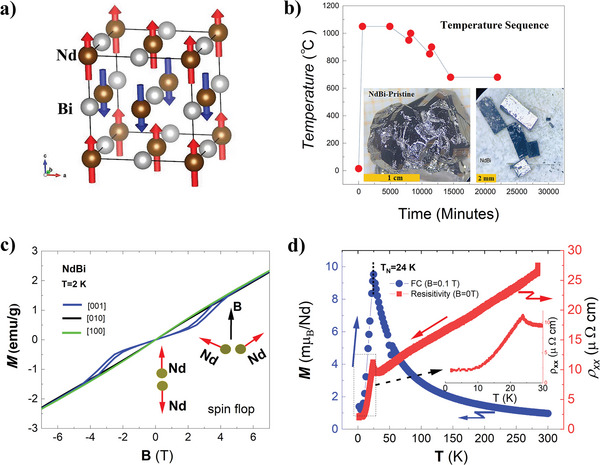
Sample growth and basic characterizations. a) The NdBi crystal structure with A‐type AFM configuration. b) The temperature sequence for the Bi‐self‐flux method. The inset shows the large pristine NdBi bulk with the scale bar of one centimeter. c) The M–H curves at 2 K, with B along ***x***, ***y*,** and ***z*** in one NdBi bulk. The spin‐flop transition with obvious hysteresis happened between 2 and 4 T along [0 0 1] direction. d) The RT and MT curves, respectively, corresponding to the right and the left axis. The insets show the zoomed‐in RT below ***T**_N_*. There are not obvious abnormal kinks or humps appearing within our measurement resolution.

Rare‐earth monopnictide NdBi has a NaCl‐type crystal structure with a space group of 225.^[^ ^39^ ^]^ The magnetic moments from Nd atoms are aligned in the type of A‐type antiferromagnet,^[^ ^40^, ^41^ ^]^ which features one ferromagnetic layer alternatively antiparallel to another layer along [0 0 1] direction, as shown in **Figure** **1a**. In order to be consistent with the coordinate system in ref. [34] we define the ***z*** along [0 0 1] direction, the ***x*** and ***y*** along [100] and [010], respectively. Figure 1b shows the temperature sequence used for the box furnace that is also described in Experimental Section. The obtained NdBi bulks and samples are shown in the inset. Figure 1c shows the 2 K M–H curve along three crystallographic directions. Noticeably, one of the M–H curves shows a sharp spin‐flop transition with obvious hysteresis between 2 and 4 T, which provides a powerful handle for adjusting the magnetic structure, as discussed in the next, may manipulate the peculiar SSs in the future thin films works, and thus attracts great attention in recent years.^[^ ^5^, ^28^, ^37^, ^38^, ^42^, ^43^, ^44^ ^]^ In NdBi, this direction is defined as ***z*** the direction [0 0 1].^[^ ^41^ ^]^ Figure 1d illustrates the field‐cooling magnetization (MT) curve under a magnetic field of 0.1 T and resistivity (RT), as plotted in blue and red lines, respectively. The sharp changes in MT and RT curves show that our NdBi bulk has a Neel temperature of 24 K. The residual resistivity ratio (RRR) of more than 13 from 250 to 2 K manifests the high‐quality of our samples (seen in the inset of Figure 1b). To be mentioned is both RT and MT do not show any abnormal characteristics such as kinks or humps below 24 K seen in the inset of Figure 1d, suggesting the high stability of the collinear antiferromagnetic structure (***1q*** magnetic vector).

### 2.2 MR Measurements at Different Temperatures and Angles

**Figure 2:**
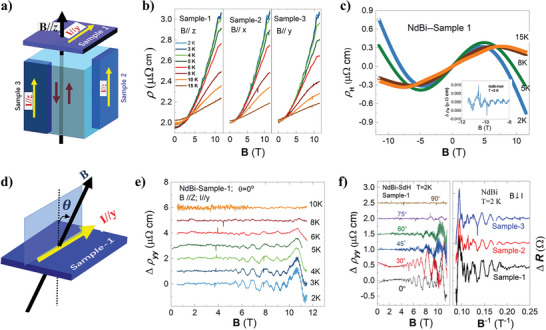
MR measurements at different temperatures and angles. a) Illustrates the configurations of Sample‐1, Sample‐2 and Sample‐3. b) The magnetoresistivity for the Sample‐1, Sample‐2, and Sample‐3. c) The Hall resistivity from Sample‐1 at different temperatures. The inset shows the Hall SdH oscillations. d) Illustrates the configuration of the MR measurements at a different angle for the Sample‐1. e) The MR‐SdH oscillations extracted from the Sample‐1 after the polynomial subtractions. f) The angle‐varying MR‐SdH oscillations for the Sample‐1 at 2 K. The angles are defined in (d).

**Figure** **2a** illustrates the three samples successively cut from one NdBi bulk along three directions, as labeled as Sample‐1, Sample‐2, and Samples‐3. Figure 2b shows the MR for the three samples with B//***x***, B//***y*** and B//***z*** at different temperatures. Clearly, there is very small MR anisotropy among them, including the amplitude of MR and the onsets of the MR‐SdH oscillations. Therefore, systematic MR measurements were mainly carried out on Sample‐1 in this study. Figure 2c shows the Hall resistivity of Sample‐1 at different temperatures. The Hall resistivity behaves nonlinearly changing with the magnetic field, which results from the *n*–*p* charge compensation since the ARPES has demonstrated both electron and hole pockets occupying a large portion at the Fermi surface.^[^ ^34^ ^]^ Figure 2e shows the residual longitudinal MR‐SdH oscillations after polynomial background subtraction. With the increasing of temperature, the amplitude of the MR‐SdH oscillations decreases rapidly and vanishes at 8 K, suggesting the decoherent scattering is strong, namely, there exists a relatively large Dingle temperature in NdBi.^[^ ^45^ ^]^ When the magnetic field is higher than 8 T, the MR‐SdH oscillations are superposed with multi‐frequencies, showing the complexity of the band structure at the Fermi surface in NdBi. Figure 2d defines the angles for Figure 2f. Figure 2f shows the MR‐SdH oscillations changed with angles at 2 K, which is crucial for the clarification of the dimensionality of each electronic band and their anisotropy along different crystallographic directions, as shown in the left and right panels, respectively.

One of the important features in NdBi is that the ARPES pattern shows twofold rotational symmetry with slight distortion,^[^ ^34^ ^]^ other than an ideal fourfold symmetry as should be in a face‐centered cubic system, which was supposed to be resulting from an unknown symmetry hidden in the magnetic structure. The right panel of Figure 2f shows the MR‐SdH oscillations for Sample‐1, Sample‐2, and Sample‐3. No obvious differences can be identified among them. However, the SdH oscillation methodology has a shortcoming in depicting the shape details of the Fermi surface.^[^ ^45^ ^]^ One might attribute the difference to the sample's orientation mismatches defined in this work and the ARPES measurements.^[^ ^34^ ^]^ In fact, we have checked the directions by XRD technique for all our NdBi samples, as shown in Figure S3 (Supporting Information). The detailed analysis on MR‐SdH oscillation is going to be shown in the next.

### 2.3 SdH Oscillations from the Dirac Surface State in NdBi

**Figure 3:**
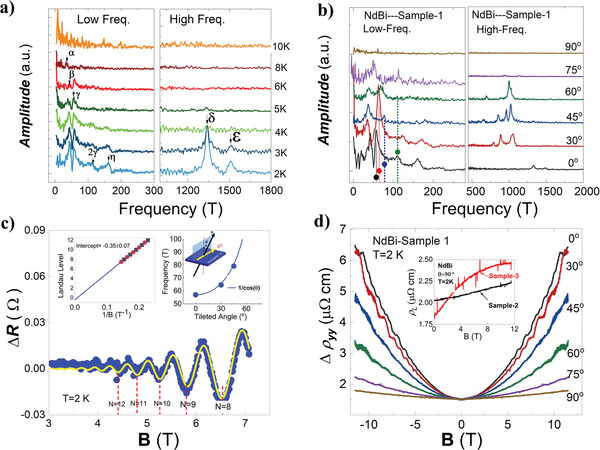
SdH oscillations from the Dirac surface state in NdBi a) The FFT on the MR‐SdH oscillations. The six FFT peaks are labeled as α, β, γ, η ,  and δ, ε. b) The angle‐varying FFT on the SdH oscillations at 2 K. The dashed lines and the filled circles guide the FFT peak of 57 T evolving with angles. The high FFT frequencies change violently, manifesting their SdH‐oscillations from the bulk. c) The low magnetic field MR‐SdH‐oscillations fit using L–K formula for 2D system with the frequency of 57 T, yielding the effective mass of 0.23 *m*_0_. The left panel of the insets shows Landau‐fan diagram where the data were collected from the Landau index from 8 to 12, yielding an intercept of −0.35 ± 0.07. In the right panel, the blue‐filled circles show the FFT peaks of 57 T changing with the tilted angle, which is well fit to the function 1/cosθ, clarifying the its 2‐dimensionality. d) The magnetoresistivity under different angles at 2 K. The inset shows the magnetoresistivity under B//I for the Sample‐2 and Sample‐3 also at 2 K.

The MR‐SdH oscillations from bulk and SSs have different angle dependencies, which thus are often used to investigate the dimensionality of electronic bands. **Figure** **3a** shows the Fast‐Fourier‐Transform (FFT) on the MR‐SdH oscillations in Figure 2e. Up to six peaks, namely four frequencies below 300 T and two frequencies higher than 1000 T, are distinguished, which are labeled as α,  β,  γ, η and δ, ε, respectively. The FFT in Figure 2f is shown in Figure 3b. The high‐frequency δ and ε vanish abruptly from 0° to 30°, while the two frequencies ≈1000 T vary slowly from 30° to 60° The high FFT frequencies exhibit typical oscillations from the bulk bands. However, the low frequency‐peaks are changing softly from 0° to 90° In the inset of Figure 3c, the projected FFT frequencies are shown. Strikingly, the frequency of 57 T, namely the γ frequency, has an obvious cosine dependence of the angles, although the amplitude decreases rapidly after 45° strongly suggesting the 2‐dimensionality of the MR‐SdH oscillations. More importantly, the frequency of 57 T just refers to the MR‐SdH oscillations happening below 8 T, as seen in Figure 3c.

Since the dimensionality of the MR‐SdH oscillations of 57 T is confirmed, we can use the Lifshitz–Kosevich (L–K) formula to investigate its topology, namely the Berry phase, and the effective mass of carriers. The generic L–K formula is expressed as:

(1) $$\begin{array}{c} \Delta \rho \propto \frac{\alpha T/\Delta E_{N}\left(B\right)}{sinh\left(\alpha T/\Delta E_{N}\left(B\right)\right)}e^{-\lambda T_{D}/\Delta E_{N}\left(B\right)}cos\left[\;2\pi \left(\;\frac{F}{B}-\frac{1}{2}+\beta +\delta \;\right)\;\right] \end{array}$$

where, the factors α  =  2π^2^ *k*_B_, Dingle temperature *T*_D_ =$\frac{h}{4\pi^{2}\tau k_{B}}$, *k*_B_ is the Boltzmann constant, *h* is the Plank constant, τ is the elastic scattering time, *F* is the frequency of a MR‐SdH oscillations and β is the Berry phase.^[^ ^46^ ^]^ The dimensionality factor δ equals to 0 and $\pm \frac{1}{8}$ for the 2D and 3D MR‐SdH oscillations, respectively. The effective mass of charge carriers m* is included in the interval of Landau levels $\Delta E_{N}\left(B\right)=\frac{heB}{2\pi m^{*}}$. After taking these constants into the Equation (1) and setting δ  =  0 for 2D system, the L–K formula can be simplified as:

(2) $$\begin{array}{ccc} \Delta \rho & & \propto \frac{1.47589T\times m_{e}^{*}/B}{sinh\left(1.47589T\times m_{e}^{*}/B\right)}e^{-1.47589T_{D}\times m_{e}^{*}/B}\\ & & \;\times cos\left[2\pi \left(\frac{F}{B}-\frac{1}{2}+\beta \right)\right]\; \end{array}$$

where, $m_{e}^{*}$ is in the unit of the free electron mass. Setting the frequency F = 57 T and fitting the curve in Figure 3c, we obtained the $m_{e}^{*}$, β and T_D_ as 0.23, 0.31 and 51 K, respectively. The nonzero Berry phase is the hallmark of Dirac fermions that have been widely studied in recent decade years in various Dirac systems such as graphene,^[^ ^47^ ^]^ topological SS in 3D topological insulators,^[^ ^48^, ^49^ ^]^ Dirac semimetals,^[^ ^50^, ^51^ ^]^ etc. In order to examine this nonzero phase whether coming from the generic quantum orbit,^[^ ^52^ ^]^ the Landau‐fan is plotted in the left panel of Figure 3c, which yields an intercept of −0.35  ±  0.07, highly consistent with the value from the L–K fit.^[^ ^48^ ^]^ One might also think this nontrivial Berry phase coming from a 3D Dirac cone when NdBi has a **2*q*** magnetic vector, as predicted in the ref. [35] This can be excluded by examining the magnetoresistivity under B//I, namely θ = 90^○^ in Figure 2d. In Dirac semimetals, the degenerate 3D Dirac cone is split into one pair of Weyl cones in a centrosymmetric crystal when external magnetic field is applied along the current.^[^ ^4^, ^53^ ^]^ Consequently, the chiral electrons are pumped from one Weyl cone to another, resulting in the decreasing of the resistivity, namely the chiral anomaly.^[^ ^54^ ^]^ Thus, the chiral anomaly induced negative magnetoresistivity (NMR) plays a crucial role in tracking Dirac/Weyl fermions.^[^ ^14^, ^22^, ^23^, ^55^, ^56^, ^57^ ^]^ In fact, as shown in Figure 3d, the magnetoresistivity under B//I in Sample‐1, Sample‐2, and Sample‐3 at 2 K does not show any negative magnetoresistivity, even though the magnetic field has increased up to 11.5 T. In Sample‐3, the MR behaves saturated at 11.5 T and may become decreasing in the high magnetic field. In this regard, we used the steady high magnetic field to investigate this phenomenon. The results are shown in Figure S7 (Supporting Information), from which no negative MR is observed. Finally, the Fermi energy can be naturally evaluated using the formula for Dirac cone SS,

(3) $$E_{F}^{Dirac}=\hbar \nu_{F}\;k_{F}$$

(4) $$F=\frac{\phi_{0}}{2\pi^{2}}A_{ext}$$

Where the ν_F_, *k*_F_, ϕ_0_, and *A_ext_* refer to the Fermi velocity, Fermi vector, quantum flux $\phi_{0}=\frac{h}{2e}$, and the area enclosed by the closed contour perpendicular to the magnetic field $A_{\mathrm{ext}}=\pi k_{F}^{2}$. Combing these relations, the Equation (3) can be simplified as:

(5) $$E_{F}^{Dirac}=\frac{0.231774\;F}{m_{e}^{*}}$$

from which the $E_{F}^{Dirac}$ was extracted as 57 meV, very close to the value evaluated from the ARPES.^[^ ^34^ ^]^

### 2.4 DFT Reproducing the Fermi‐Arc and Dirac Surface States in NdBi

**Figure 4:**
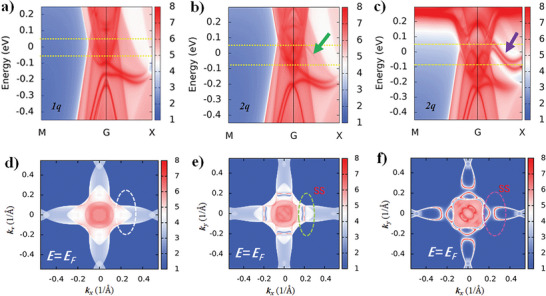
DFT reproducing the peculiar surface states. a) Shows the surface spectral functions between the high symmetry points μ − Γ − X, using the first method and the *1q* magnetic vector. The color bar stands for the density of states. b,c) show the surface spectral functions between the high symmetry points μ − Γ − X with the magnetic vector ***2q***. The different surface states, shown by the green arrow in (b) and the purple arrow in (c), are obtained by the first method and second method, respectively. d–f) are the surface spectral functions for ***E_F_*** = 0 meV in (001) surface. The dashed ellipses indicate the gradual emergence of the surface states. Here, the ***E_F_*** is the DFT calculated value.

Reproducing the peculiar SSs by DFT calculation is helpful for us to understand the close relations between the electronic bands and the magnetic properties in NdBi. Inspired by the recent work,^[^ ^35^ ^]^ we performed DFT calculation with a different strategy, and the details are described in Experimental Section. We found the calculations converge in two significantly different ways: one state with a small orbital magnetic moment (1.5 µB) of Nd, and the other state with a larger orbital magnetic moment (5.8 *µ*B). The latter state is unfavorable in total energy, but its 2 ***q*** surface spectral function is consistent with the result of ARPES^[^ ^35^ ^]^ It indicates the orbital magnetic moment from Nd plays a crucial role in fostering the peculiar SSs. **Figure** **4a** shows the surface spectrum function with the magnetic vector of ***1q***, as determined by the neutron scattering experiments.^[^ ^40^ ^]^ Indeed, the peculiar SSs do not appear along the high symmetry points Γ‐X,^[^ ^34^ ^]^ as shown in Figure 4d, which manifests the ***1q*** magnetic structure does not support the appearance of the peculiar SSs in NdBi. In Figure S8a (Supporting Information), the disappearance of the SSs is also shown by the stacking plot of the constant energy contours, ranging from *E*_F_+0.05 to *E*_F_−0.08 eV. Figure 4b shows the surface spectrum function with a small orbital magnetic moment and magnetic vector of ***2q***, as defined in the ref. [35] Interestingly, a SS band emerges along Γ‐X, as shown in Figure 4b,e, is guided by the green arrow and the dashed green ellipse, respectively. Unfortunately, this SS does not match with the reported ARPES at all.^[^ ^34^ ^]^

Figure 4c shows the surface spectrum function with a large orbital magnetic moment and ***2q*** magnetic structure. Strikingly, the SSs show up with two branches indicated by the purple arrow. In Figure 4f, the two branches behave a Fermi‐arc curved toward the Brillouin zone center Γ and a droplet‐like contour distributes long Γ‐X, which have a very high similarity with the reported ARPES results.^[^ ^34^ ^]^ With the change of the cutting‐energy, the droplet‐like contour is gradually shrinking into a point at *E*_F_−0.08 eV, forming a cusp‐like band structure (shown in Figure S8c, Supporting Information). One might also note in Figure 4f that the droplet‐like Dirac cones have slight distortions exhibiting a twofold symmetry and hence might result in differences in the MR‐SdH oscillations along ***x, y***, and ***z*** directions. In fact, the frequency of the MR‐SdH oscillations is only determined by the area enveloped by the closed contour in momentum space, regardless of the shape of the area in details^[^ ^46^ ^]^ In this regard, our MR‐SdH oscillations exhibit fourfold symmetry, as shown in Figure 2f and Figure S6 (Supporting Information), just right show the basic features of the Dirac SSs in the DFT calculations. In combination with our MR‐SdH results and the area enclosed by the droplet‐like contour, we confirm the Fermi energy in our samples to be *E*_F_−0.04 eV (shown in Figure S8c, Supporting Information).

## 3 Conclusion

In conclusion, the SdH oscillation for the Dirac surface state in NdBi is successfully identified, and the 2D Dirac cone accompanying the Fermi‐arcs is also well reproduced by our DFT calculations with the ***2q*** magnetic structure, which is of essential importance to the advanced AFM spintronics devices innovations. The sharp spin‐flop transition with obvious hysteresis, the first example in the most recently studied rare‐earth monopnictide,^[^ ^34^, ^58^, ^59^ ^]^ may provide a powerful handle for controlling the peculiar SSs. Our DFT calculations reveal the larger orbital magnetic moment of Nd plays a crucial role in fostering the peculiar SSs, which enables X‐ray Magnetic Circular Dichroism (XMCD) spectroscopy to be a powerful tool for unveiling the mystery of the peculiar band‐splitting effect in NdBi^[^ ^60^, ^61^ ^]^ Our study may provide important guidance for the development of new antiferromagnet‐based spintronics devices based on cutting‐edge rare‐earth monopnictide NdBi.

## 4 Experimental Section

### Crystal Growth and Characterizations

The high‐quality single crystalline NdBi was grown by the self‐flux method, using bismuth as the flux. The starting atomic ratio of Bi:Nd was 84:16. The high‐purity grains were put in a crucible and then inserted into a quartz tube. After a long period of pumping and three times argon gas flushing, the quartz was sealed with quartz wool used as the flux filter. The zigzag‐type temperature sequence was set for a box furnace. The furnace was first heated up to 1050 °C within 600 min and followed by 4320 min of temperature maintenance for sufficiently homogenizing. In the following, the molten liquid was cooled to 950 °C within 3000 min and then heated up to 1000 °C within 300 min. After that, it was decreased to 850 °C and went up to 900 °C. Finally, it was slowly cooled to 680 °C and then was kept for 3 days for annealing. After the flux was centrifuged, the pristine NdBi bulk in a size of 2 cm  ×  1 cm  ×  1 cm was obtained, as shown in the inset of Figure 1b. Because of the high sensitivity to the air, the samples’ preparation had to be completed within 15 min, including the electrode fabrication using AB epoxy and was inserted into the chamber of a liquid‐helium‐free superconducting measurement system. To protect from air contamination during the XRD measurements, the NdBi powder was made in a glove box filled with high‐purity argon gas. The plastic thin film was covered using the N‐grease as the encapsulation adhesive. Therefore, the XRD peaks in the angle from 20° to 23° were coming from the plastic film, as shown in Figure 1c and Figure S2 (Supporting Information). The high field magnetoresistivity and magnetization measurements were carried out in the national steady high magnetic field center in Hefei, China. on the facility of WM5.

### Density Functional Theory (DFT) Calculations

Spin‐polarized density functional theory computations were performed using the Vienna ab initio simulation (VASP) package,^[^ ^61^ ^]^ with a generalized gradient approximation (GGA) using the Perdew–Burke–Ernzerhof exchange‐correlation functional including spin‐orbit coupling (SOC),^[^ ^62^ ^]^ and a GGA + U strategy to describe the strong Coulomb interaction between the half‐filled 4*f*‐shells of Nd. The onsite Hubbard parameters U and J were set to 6.3 and 0.7 eV, as used in ref. [63]. The experimental lattice constant of 6.41 Å, and a Γ‐centered 13 × 13 × 13 k mesh sampling for the Brillouin zone with a kinetic energy cutoff of 300 eV were used. The band structures of the anti‐ferromagnetic NdBi bulk with ***1q*** and ***2q*** wave vectors were reproduced by maximally localized Wannier functions implemented in the WANNIER90 package,^[^ ^64^ ^]^ including SOC within [*E*_F_−5 eV, *E*_F_+1 eV] with Nd *s*‐*d*‐*f* and Bi *p* orbitals. Then the surface spectral function was calculated with the surface Green's function methods by Wannier Tools package.^[^ ^65^ ^]^ Due to the difference in the details of the GGA+U+SOC calculation, two kinds of magnetic moment results in anti‐ferromagnetic ***2q*** NdBi bulk were obtained. The first method: two‐step SOC calculation. The non‐SOC calculation was performed first, and then the wave function was read for SOC calculation. For each Nd atom, the calculated orbital magnetic moment and total magnetic moment were 1.5 and 3.0 μ_*B*_, respectively. The total energy obtained by this method was 2.0 eV/(Nd_4_Bi_4_) cell lower than that of the second method, but the surface spectral function was inconsistent with the result of ARPES.^[^ ^34^ ^]^ The second method: one‐step SOC calculation. The calculated orbital magnetic moment and total magnetic moment were 5.8 and 3.0 μ_*B*_ per Nd atom. Although this electron structure with a larger Nd orbital magnetic moment was predicted to have a higher free energy, the surface spectral function was close to the results of ARPES, as reported in ref. [35]

## Conflict of Interest

The authors declare no conflict of interest.

## Author Contributions

R.Q.W., J.C.Z., T.L., and K.M.C. contributed equally to this work. T.S.C., S.J.Y., and J.L.W. conceived and supervised this project. T.S.C. designed the experiments, R.Q.W., J.C.Z., T.L., C.Y.X., and Z.Y.L. performed the magnetotransport experiments and analyzed the data. The magnetization was measured by L.S.L.. S.J.Y. performed the DFT calculations. R.Q.W. performed the Scanning electron microscopy and energy dispersive X‐ray spectroscopy (SEM‐EDS) measurements and analysis. T.S.C., S.J.Y., and J.L.W. drafted the paper. All authors discussed the results and commented on the paper.

## Supporting information

Supporting Information

## Data Availability

The data that support the findings of this study are available from the corresponding author upon reasonable request.
