# Supplementary material for: SdH Oscillations from the Dirac Surface State in the Fermi‐Arc Antiferromagnet NdBi
Source: Adv Sci (Weinh). 2023 Oct 25;10(35):2303978. doi: 10.1002/advs.202303978 (PMC10724392; doi:10.1002/advs.202303978)
Supplement: Supplementary file 1 — Supporting Information [file ADVS-10-2303978-s001.pdf]

ADVANCED SCIENCE
Open Access

Supporting Information

for *Adv. Sci.*, DOI 10.1002/advs.202303978

SdH Oscillations from the Dirac Surface State in the Fermi-Arc Antiferromagnet NdBi

Ruoqi Wang, Junchao Zhang, Tian Li, Keming Chen, Zhengyu Li, Mingliang Wu, Langsheng Ling, Chuanying Xi, Kunquan Hong, Lin Miao, Shijun Yuan, Taishi Chen* and Jinlan Wang**

Supporting Information

SdH oscillations from the Dirac Surface State in the Fermi-arc Antiferromagnet NdBi

Ruoqi Wang, Junchao Zhang, Tian Li, Keming Chen, Zhengyu Li, Mingliang Wu, Langsheng Ling, Chuanying Xi, Kunquan Hong, Lin Miao, Shijun Yuan, Taishi Chen*, Jinlan Wang**

R. Wang, Dr. J. Zhang, K. Chen, Dr. M. Wu, Prof. K. Hong, Prof. L. Miao, Prof. S. Yuan, Prof. T. Chen, Prof. J. Wang
Key Laboratory of Quantum Materials and Devices of Ministry of Education, School of Physics, Southeast University, Nanjing 211189, China

T. Li, Z. Li, L. Ling, C. Xi
High Magnetic Field Laboratory, Chinese Academy of Sciences, Hefei, 230031, China.

E-mail: chentaishi@seu.edu.cn, siesta@seu.edu.cn, jlwang@seu.edu.cn

Table of Contents:

1. Information text 1: The XRD measurement on NdBi.
2. Information text 2: The magnetization characterizations on NdBi.
3. Figure S1: The photography of the freshly made samples on the puck and a destroyed NdBi sample after one week of staying in air.
4. Figure S2: The confirmation of the low-angle impurity peaks in XRD pattern
5. Figure S3: The XRD results on bulks of NdBi single crystal.
6. Figure S4: Curie-Weiss's law fit the MT curve and high magnetic field investigation of the magnetization.
7. Figure S5: SEM-EDS analysis on a freshly cleaved NdBi sample.
8. Figure S6: The MR-SdH oscillations and FFT on Sample-1, Sample-2 and Sample-3.
9. Figure S7: The magnetoresistivity under the configuration of B//I at 2 K.
10. Figure S8: Spectral functions of NdBi (001) surface cut at different energy.
11. Figure S9: The DFT band structures obtained by the two methods.
12. Figure S10. The DFT band structures for ferromagnetic NdBi.
13. Figure S11. The Fermi surface for different magnetic structure of NdBi.

Information text 1: The XRD measurement on NdBi

Figure S2A shows the XRD results on the NdBi powder and the rectangle bulks, where the low-angle diffraction is from the covered plastic film used for protection from air corruption. All the peaks match well with the simulated pattern, which yields a lattice constant of a=6.42657Å and is consistent with the previous works [34]

To verify the low angle peaks not from the NdBi powder, we carried out the XRD measurements shown in Figure S2B in three steps, namely the first step on pure plastic film as illustrated by

the black line. In the second step, the N-greased was added, and the results are shown by the red line, from which we confirmed the low-angle peaks come from the plastic film.

Because of the high sensitivity to the air, the samples' preparation had to be completed within 15 minutes, including the fabrication of the electrodes using AB epoxy and being inserted into the chamber of a liquid-helium-free superconducting measurement system. To protect from the air contamination during the XRD measurements, the NdBi powder was made in a glove box filled with high-purity argon gas. The plastic thin film was covered using the N-grease as the encapsulation adhesive. Therefore, the XRD peaks in the angle from 20 deg. to 23 deg. are coming from the plastic film, as shown in Figure 1c and Figure S2. The high field magnetoresistivity and magnetization measurements were carried out in the national steady high magnetic field center in Hefei, China. on the facility of WM5.

Information text 2：The magnetization characterizations on NdBi

In this study, we characterized the magnetization for NdBi on two facilities: the low-field M(B) and MT on a MPMS, the high-field M (B) on the Steady High Magnet Field Facility (SHMFF). The results are shown in Figure S5 and Table 1. In Figure S4A, the field-cooling susceptibility at 0.1 T is shown. The Curie-Weiss formula $\chi = \frac{C}{T-\theta_{C-W}}$ was used to fit the MT curve between 100 K and 300 K, which yields the Curie-Weiss constant and Curie-Weiss temperature of 1.631 K·emu/mol·Gs and -1.7 K, respectively. Consequently, we figure out the effective magnetic moment of neodymium, μ_{eff}=$\sqrt{8C}$=3.61 $\boldsymbol{\mu_B}/Nd$. With the increasing of the external magnetic field, the magnetic moment increased rapidly at 16.5 T and saturated at a value of 3.267 μ_B/Nd seen in Figure S4B. The plateau between 16.7 T and 16.9 T suggests a metastable magnetic structure existing before saturation. To be noted is that the saturation magnetic moment of Nd atom is smaller than the calculated value based on the Hund's rule method $\mu_{cal} = 3.62\boldsymbol{\mu_B}$, $\mu_{cal} = g_{\boldsymbol{J}}\sqrt{\boldsymbol{J}(\boldsymbol{J}+1)}\boldsymbol{\mu_B}$, with the total angular momentum quantum number $\boldsymbol{J}$=9/2, which suggests the crystal electric field plays a substantial role in NdBi [63].

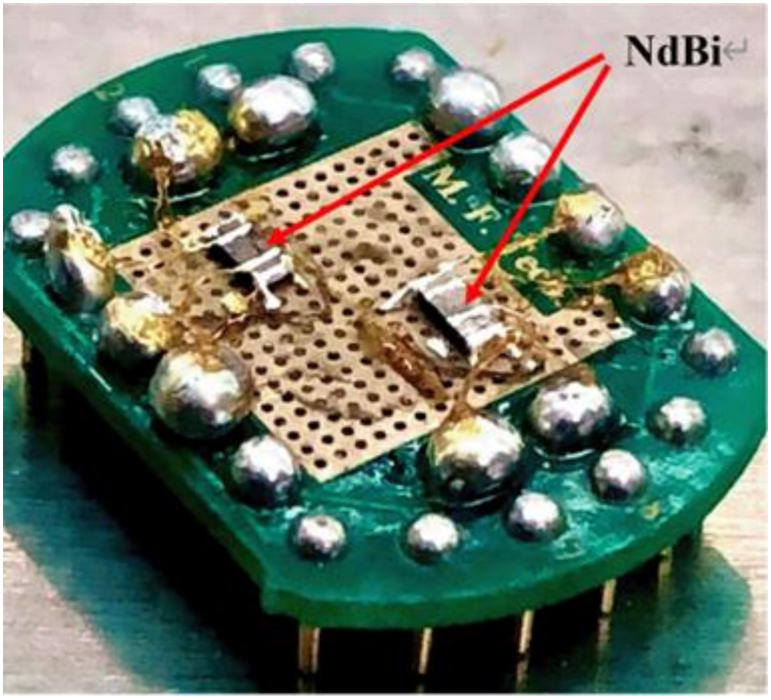

Figure S1. The photography of the freshly made samples on the puck.

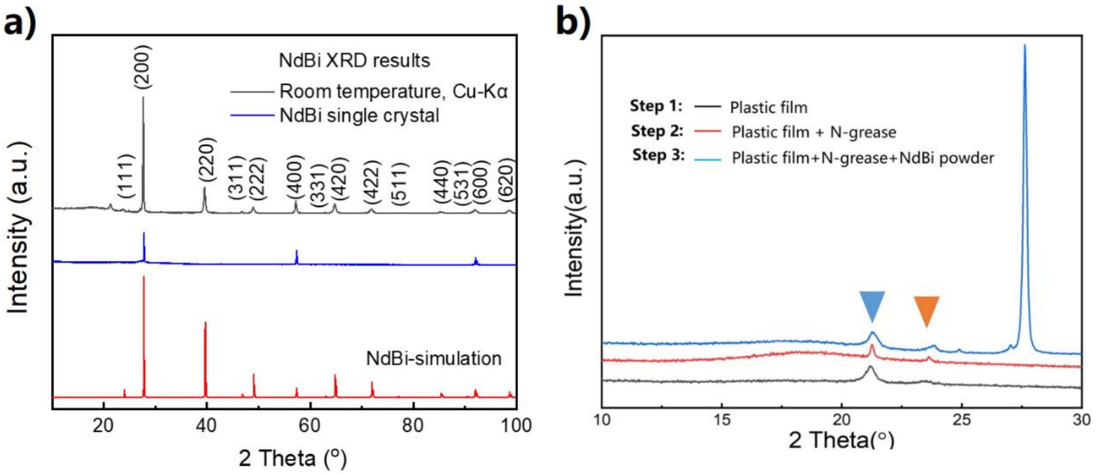

Figure S2. The confirmation of the low-angle impurity peaks in XRD pattern. a) shows the XRD characterization on the NdBi powder and single crystal surface, as plotted by the black and blue lines, respectively. The bottom red line is the simulated XRD curve using the standard CIF file from the database. **b)** verifies the low-angle XRD peaks coming from the pure plastic film.

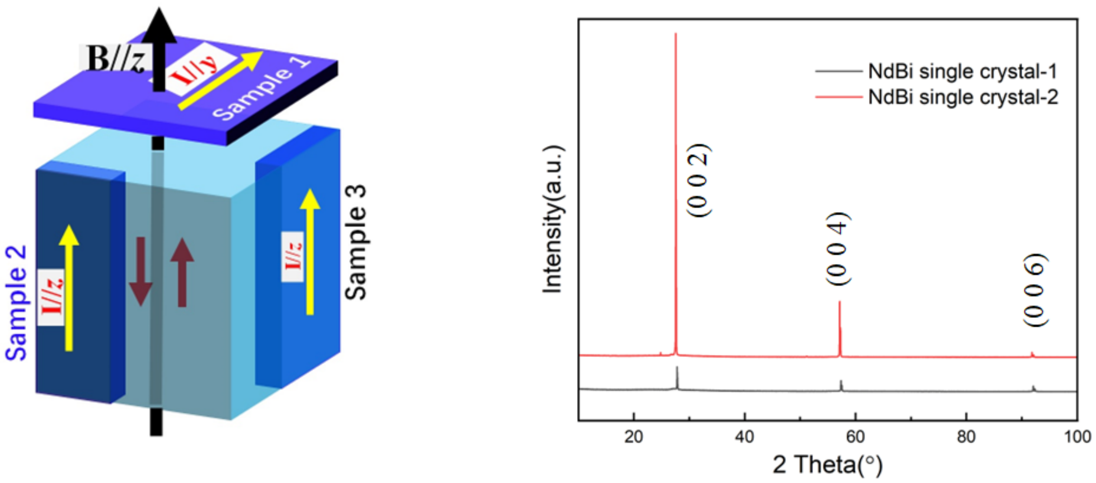

Figure S3. The XRD results on bulks of NdBi single crystal.

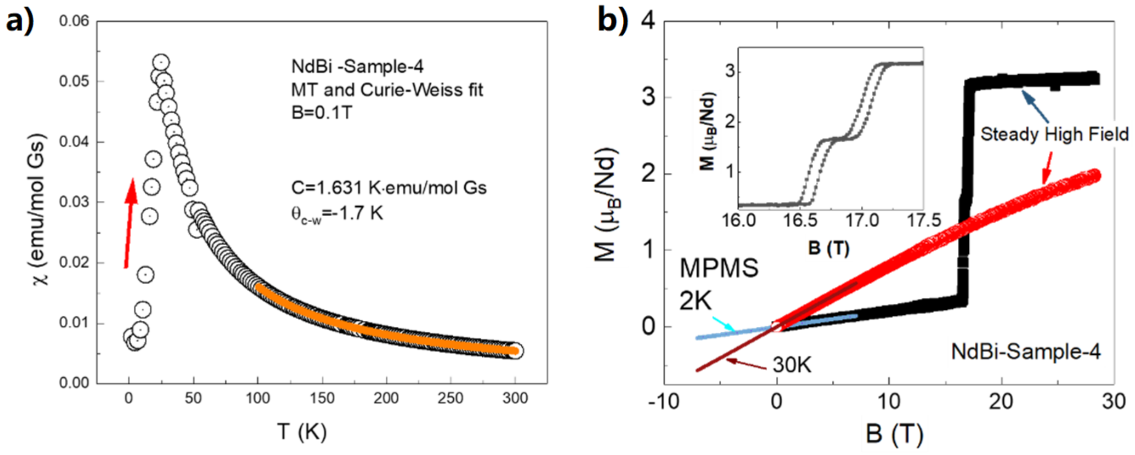

Figure S4. Curie-Weiss's law fit the MT curve and high magnetic field investigation of the magnetization.

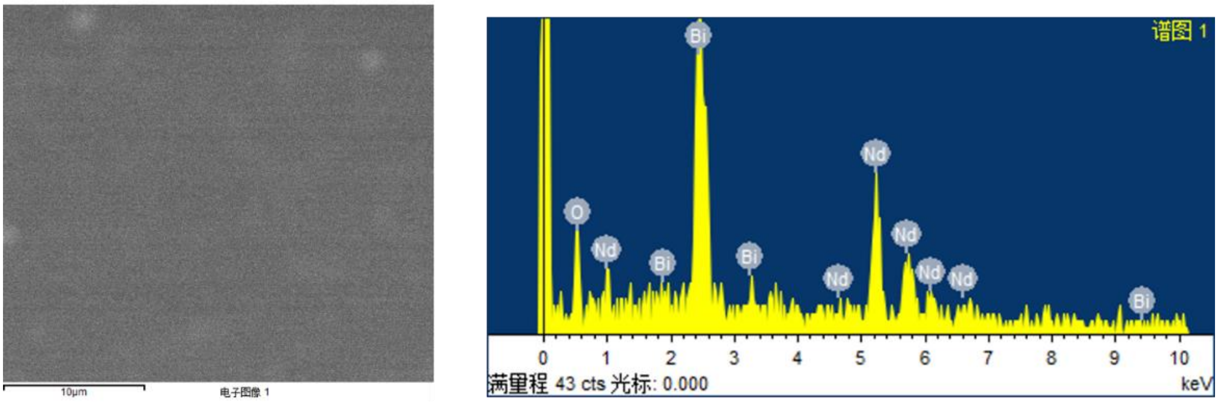

Figure S5. SEM-EDS analysis on a freshly cleaved NdBi sample.

Table 1. SEM-EDS results on the pristine NdBi single crystal.

Elements	Weight [%]	Atomic [%]
Nd L	39.58	48.8
Bi M	60.42	51.2
Totals	100.00	100.0

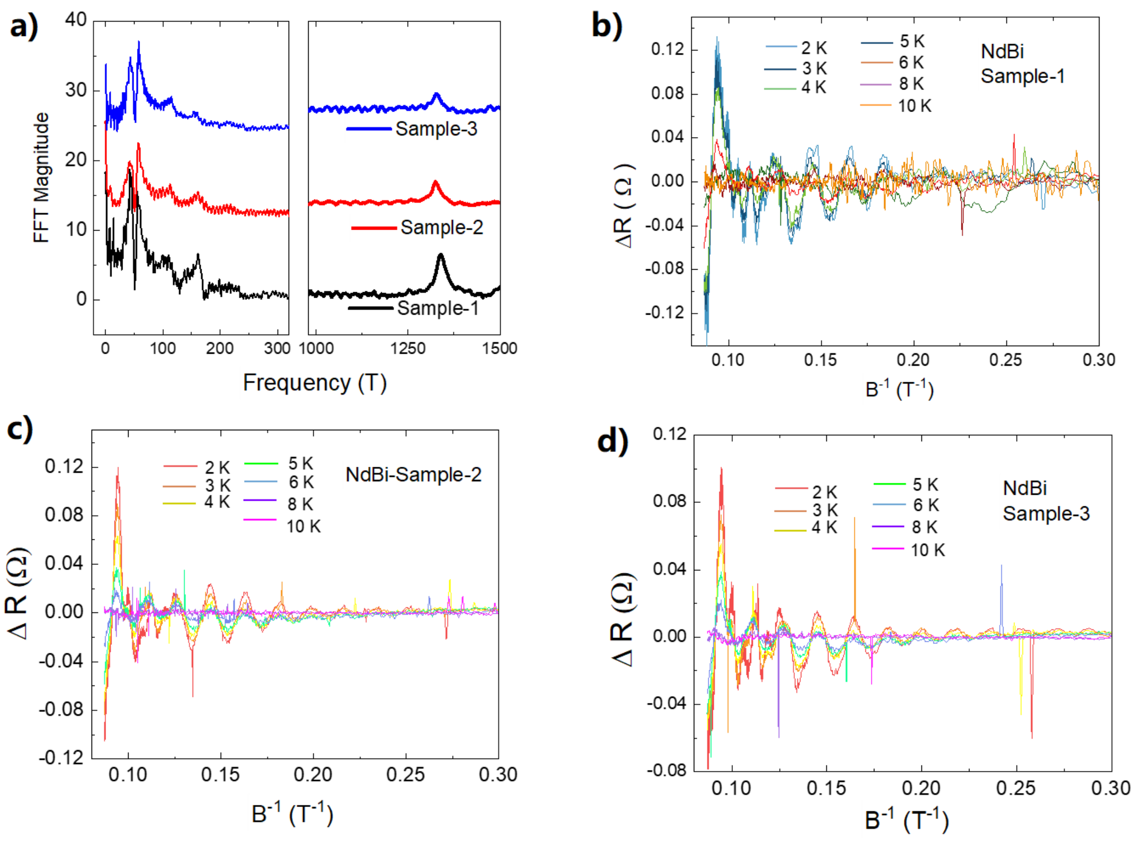

Figure S6. The MR-SdH oscillations and FFT on Sample-1, Sample-2 and Sample-3.

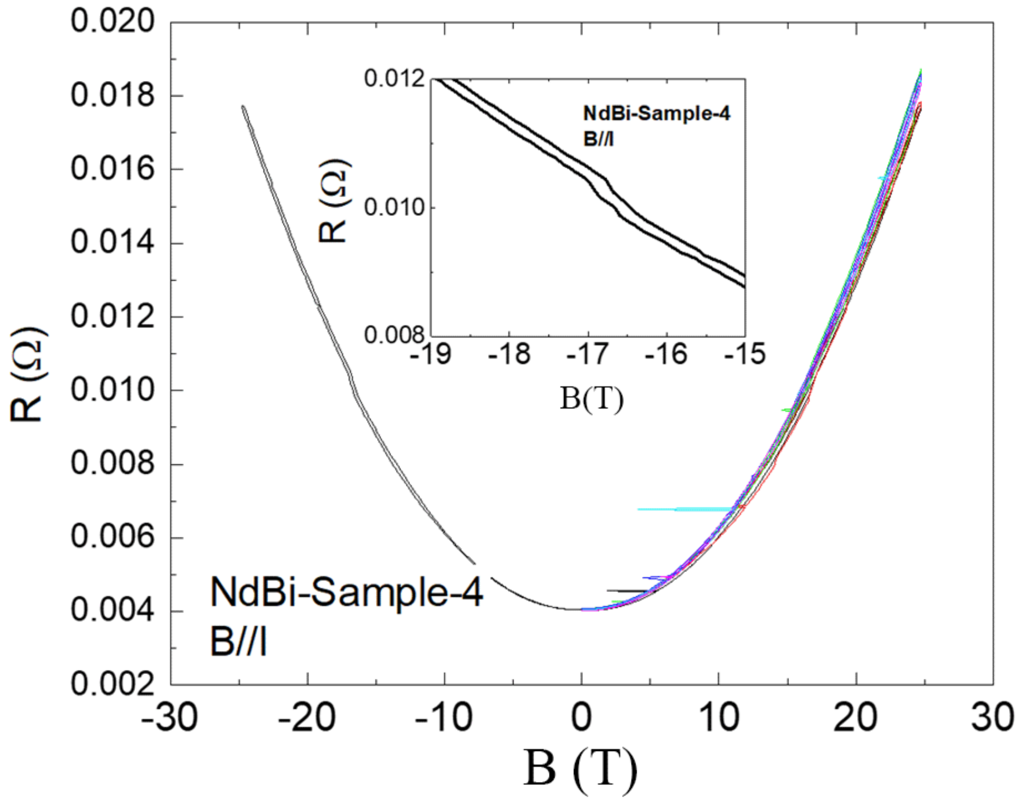

Figure S7. The magnetoresistivity under the configuration of B//I at 2 K.

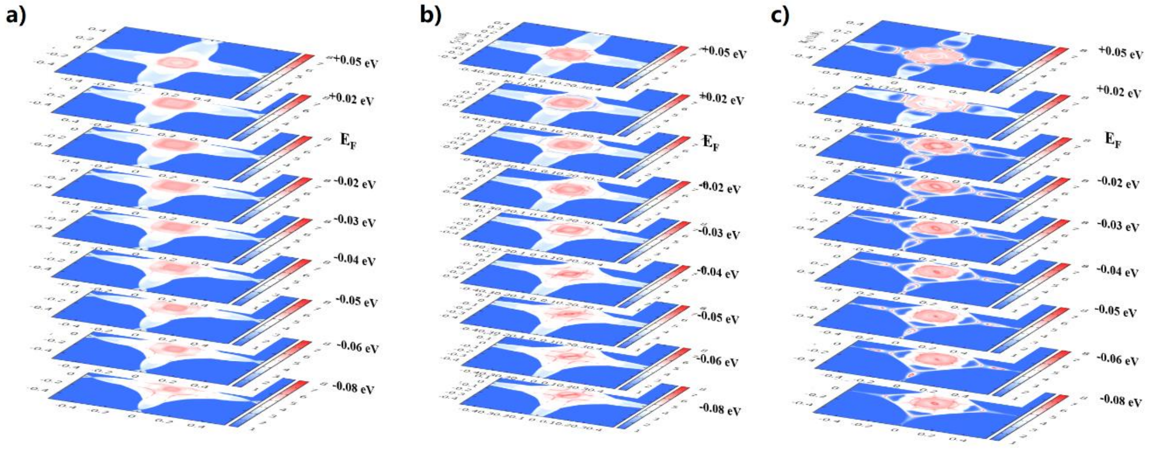

Figure S8. Spectral functions of NdBi (001) surface cut at different energy. a) and **b)** are obtained using the first method with the orbital magnetic moment of -1.5 $\boldsymbol{\mu_B}$. **a)** and **b)** correspond to the spectral functions in Figure 4a and 4b in the main text, respectively. **c)** The spectral functions obtained by the second method and refers to Figure 4c in the main text, with the orbital magnetic moment 5.8 $\boldsymbol{\mu_B}$.

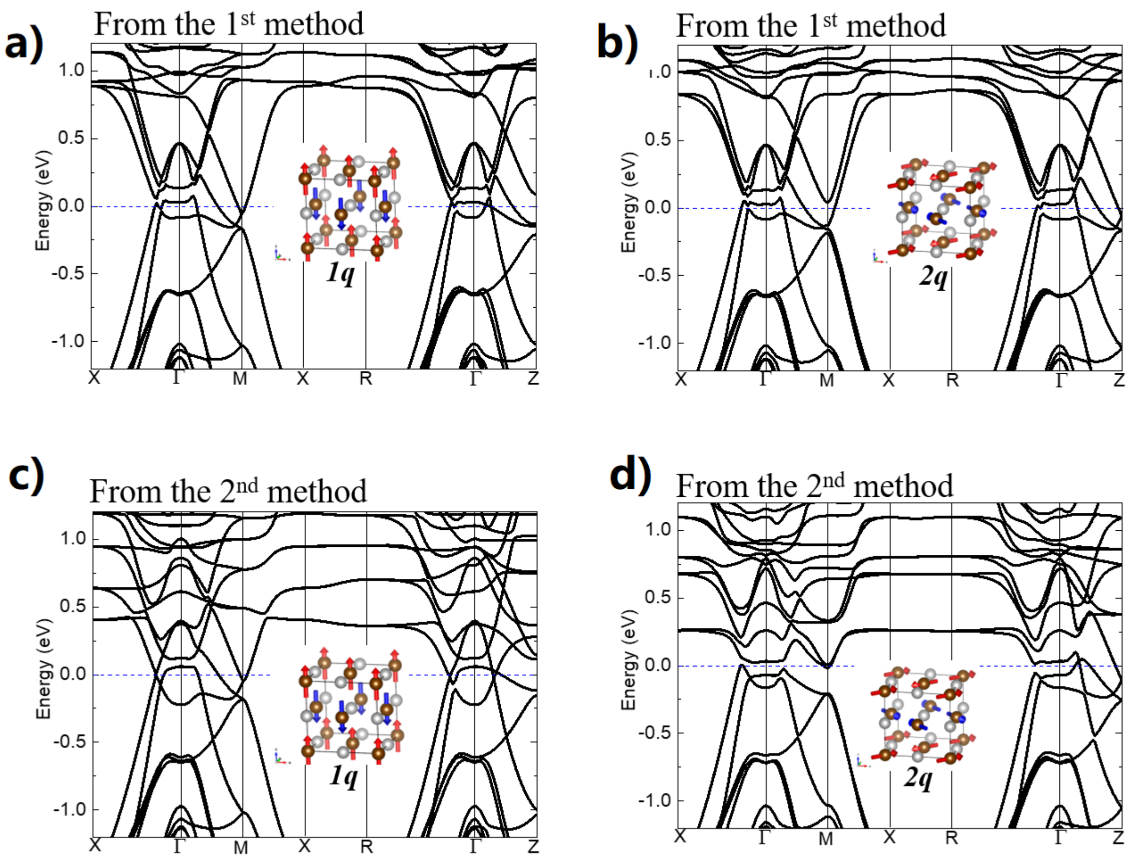

Figure S9. The DFT band structures obtained by the two methods. a) and **b)** The band structures were obtained by the first method with the orbital magnetic moment and the spin magnetic moment -1.5 $\boldsymbol{\mu_B}$ and 4.5 $\boldsymbol{\mu_B}$, respectively. The insets show the magnetic order for the two band structures. **c)** and **d)** the band structures were obtained by the second method with the orbital magnetic moment and spin magnetic moment 5.8 $\boldsymbol{\mu_B}$ and -2.8 $\boldsymbol{\mu_B}$ per Nd atom. The magnetic order for the two band structures are shown in the insets.

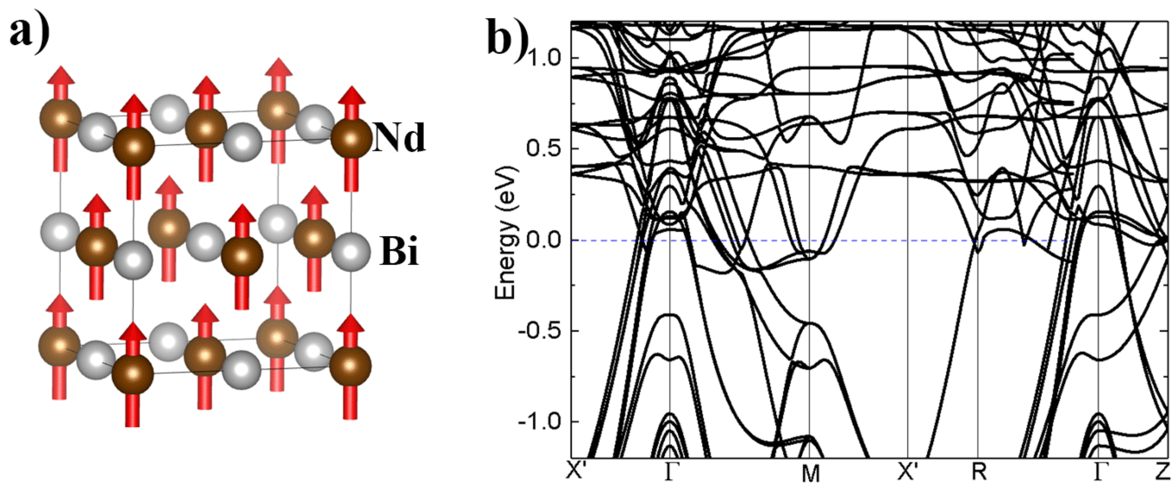

Figure S10. The DFT band structures for ferromagnetic NdBi. a) and **b)** represent the magnetic structure and the band structure, respectively.

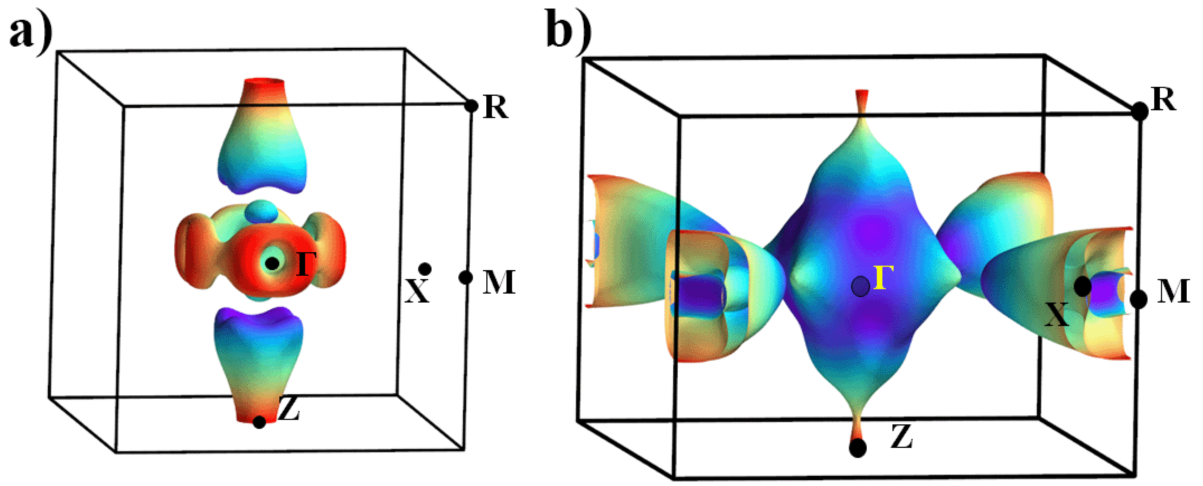

Figure S11. The Fermi surface for different magnetic structure of NdBi. a) shows the Fermi suface for 2***q*** antiferromagnetic structure at E_f=57meV, and **b)** is for ferromagnetic structure at E_f=57meV.
